# A Randomised Controlled Experimental Study on the Influence of Patient Age on Medical Decisions in Respect to the Diagnosis and Treatment of Depression in the Elderly

**DOI:** 10.1155/2009/475958

**Published:** 2010-02-16

**Authors:** Michael Linden, Guido Kurtz

**Affiliations:** ^1^Research Group Psychosomatic Rehabilitation, Charité University Medicine, Lichterfelder Allee 55, Teltow, 14513 Berlin, Germany; ^2^Department of Behavioral Medicine, Rehabilitation Center Seehof, Lichterfelder Allee 55, Teltow, 14513 Berlin, Germany

## Abstract

*Background*. Elderly patients are often treated differently than younger patients, even when suffering from the same disorder. *Objective*. The study examines the influence of “patient age” on the perception of symptoms and conclusions of physicians in respect to diagnosis and treatment. *Methods*. In a randomised controlled experimental study on medical decision-making, 121 general practitioners were given two case vignettes which contained all the criteria for major depression according to ICD-10, but differed in respect to the age of the patient (39 or 81). Reaction time, diagnostic conclusions and therapeutic recommendations were assessed by computer. *Results*. Depression and anxiety were significantly seen as more probable in the young cases and dementia and physical illness in the old. In young age, psychotherapy, pharmacotherapy and referral to a specialist or inpatient treatment were significantly more recommended than in old age, for whom supportive counselling was significantly more recommended. The time needed for a decision was significantly longer in the older patients. *Conclusion*. Ageing stereotypes can also form medical illness concepts and have a significant influence on diagnostic and therapeutic decisions.

## 1. Introduction

Elderly patients are often insufficiently diagnosed and treated. An example is the under-recognition and undertreatment of depression [1–3]. One explanation is that the diagnosis and treatment in the elderly is more complex than in younger patients because they are typically multimorbid and suffer many burdens, which makes it difficult to diagnose and treat mental as well as somatic disorders [4–6]. Symptoms which are specific diagnostic signs in younger years can become unspecific in the elderly [7]. Another explanation is that negative ageing stereotypes and emotional reactions to old age can lead to differences in the perception of elderly persons and their care [8–13]. This means that age can contribute to problems in the diagnosis and treatment of depression of elderly patients.

Information on medical decision-making in the diagnosis and treatment of the elderly is rather descriptive if not anecdotal, and there is a lack of experimental research [14]. The objective of this study is to test experimentally the influence of ageing stereotypes and of information complexity of medical decisions on the diagnosis and treatment of depression. General practitioners were given case vignettes that were identical in content but differed in respect to the age of the patient. Diagnoses and treatment recommendations were assessed. In addition, the time needed for a decision was measured, which is an indicator of the complexity of the information and the ongoing decision.

## 2. Materials and Methods

121 general practitioners (55.7% females, age 49.5 ± 8.3 years, 21.2 ± 9.5 years as physicians, 773 ± 265 patients in their practice, 35% of patients above the age of 70) from private practices were each given two case vignettes describing a female patient and mentioning all the criteria of major depression according to ICD-10:

(i) An 81(39)-year-old female patient, who has not seen her doctor for some while, is accompanied by her daughter and complains that she lost her husband five years ago and does not want to live without him. She feels a burden to everybody. She suffers from rheumatism and has difficulties moving. She regularly takes on analgesic. When asked she says she has sleep problems and that the day is a burden. She has problems with her memory and the ability to concentrate. She feels tired, worn out, and overtaxed. The daughter says that her mother has changed very much. She is withdrawn, no longer comes to see her, and no longer shows any initiative. She does not eat regularly and has lost weight. She does not know what to do with her.

(ii) An 81(39)-year-old female patient, who has not seen her doctor for some while, is accompanied by her sister. The patient complains that she was divorced some time ago and has difficulties living alone. She no longer enjoys life and feels tired and worn out. The patient suffers from hypertension and is being treated with antihypertensive drugs. As the patient is rather taken aback, the sister reports that the patient has recently become more and more disagreeable, has no initiative, and is socially withdrawn. She says that she no longer leaves the house and has lost weight. She forgets dates and has problems concentrating. When asked, the patient says that she feels sorry that she is such a burden to her family and that this causes her sleepless nights.

The investigation was undertaken in the office of the physicians. Cases were presented and answers and evaluations assessed with a laptop computer. All physicians evaluated both case vignettes, which were presented either as an 81-year-old or 39-year-old patient. The sequence of presentation of the cases and age per case were randomised by computer. Physicians were then asked to rate the case using a six-step Likert scale (very unlikely, unlikely, somewhat unlikely, somewhat likely, likely, very likely) for three factors: (a) how probable they thought this patient was suffering from an organic brain disorder/dementia, anxiety disorder, depression, or somatic illness; (b) how much they thought this patient was cognitively impaired, anxious, depressed, or somaticly ill; and (c) whether they would recommend and expect good results from pharmacotherapy, counseling, psychotherapy, somatic treatment, referral to a psychiatrist, or inpatient care.

The use of a laptop computer allowed to measure the reaction times, that is, the time between the presentation of each question and the physician's answer. This paradigm comes from studies on information processing in humans [12]. The longer the time needed to answer a question or make a rating, the more consideration is needed and the more complex the problem which has to be solved.

Statistical analyses were done with the Statistical Package for Social Sciences (SPSS). As the hypothesis was that there are differences between old and young cases per physician, the *t*-test for dependent variables was used to compare reaction times and ratings.

## 3. Results

The ratings show that in both cases depression is seen as most probable (4.69 and 4.50 for old and young cases, resp.; Table 1). But there are significant differences for differential diagnoses. Dementia (1.99 versus 3.63) and somatic illness (2,40 versus 3.40) were preferably taken into account in the older patients and anxiety disorders (2.99 versus 2.74) in the younger patients.

**Figure 1:**
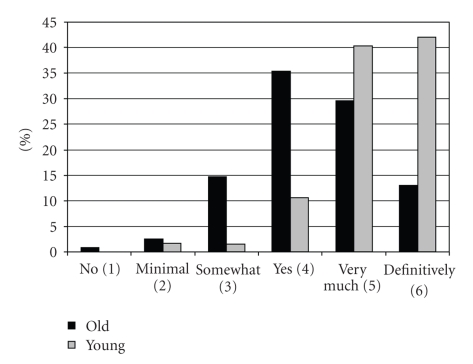
Global rating on treatment necessity.

There are also significant differences between the younger and older patients for treatment options. Referral to a psychiatrist, referral to inpatient treatment, pharmacotherapy, and psychotherapy are all more recommended in younger patients, whereas supportive counselling is seen as more appropriate in the elderly. Figure 1 shows the global rating on the necessity of treatment. The need for treatment is rated as significantly lower for older patients (4.36 versus 5.52).

**Figure 2:**
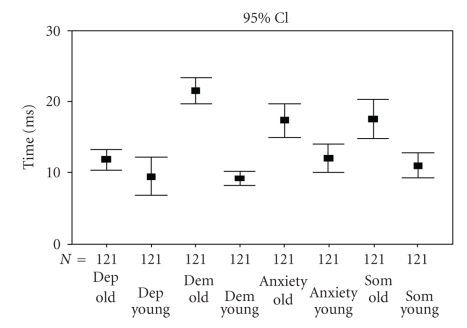
Time to answer (milliseconds).

Figure 2 shows the time needed in milliseconds to give an answer. In the elderly, all diagnostic decisions need more time to come to a conclusion regarding depression, dementia, anxiety, or somatisation (*t* = 3.9–13.3; *P* < .001). The difference is most pronounced for dementia. The information on old age with depression-like multiform symptoms makes physicians think longer.

## 4. Discussion

Before interpreting the results, some limitations of the study must be taken into account. We did not study what the physicians actually did with the older and younger patients in their daily practices. Instead, we worked with case vignettes, which contained only limited information so that physicians had to make guesses and could not clarify the cases or ask for additional information. This allowed us to study attitudes but not necessarily behaviour. Physicians also knew that they were participating in a scientific study and this might have changed their behaviour. Finally, 121 general practitioners are a large number, but the sample is still not epidemiologically representative. Results could be different for other samples.

Given these precautions, the first result of this experimental study confirms other reports by showing that there are differences in the way patients with depressive disorders are diagnosed and treated depending on age [1, 2, 8, 12, 13, 15]. When suffering from identical symptoms, different diagnoses are made in older and younger patients and, more importantly, different treatments are recommended. Because the need for treatment is significantly more questioned in older patients (Figure 1), this can be seen as an indicator of greater pessimism in respect to the elderly. This can become a problem because the treatment of depression in the elderly is as effective as in younger patients and should not be withheld because of age [16]. One could argue that complaints about memory impairment or diagnoses such as dementia and somatic illness are characteristic for the elderly. However, younger patients are not excluded from multiple infact dementia, hypertensive encephalopathy, and other illnesses of the brain or somatic illnesses. Both case vignettes also contained information in this direction.

The second result is that age produces cognitive schemata in physicians which by themselves lead to relevant differences in the care for the elderly. Our methodology allows us to study cognitive biases, that is, stereotypes. We did not ask for a categorical diagnosis but for probabilistic judgements, which is especially appropriate in the elderly [17]. As the case vignettes were identical in respect to presentation, type, complexity, and severity of symptoms, it can be concluded that information on age was sufficient to lead to different diagnostic and therapeutic medical conclusions. Information about age obviously sets another frame of reference for the interpretation of identical signs and symptoms.

The third result is that this age-dependent frame of reference leads to differences in information processing. Physicians need significantly longer to make up their minds when confronted with an elderly person. This can be interpreted as an indicator of greater information complexity and difficulty of the decision-making process [18, 19]. From the literature, it is known that the same symptoms can have different meanings in people of different ages. There is much more ambiguity in the meaning of illness signs under conditions of old age and multimorbidity [7, 20].

## 5. Conclusions

The results from this randomised controlled experimental study show that age of patient stimulates age stereotypes or cognitive schemata in physicians, and that this has a great impact on medical decisions. Guidelines for the treatment of the elderly and education of carers should take into account the nature and effects of ageing stereotypes [21, 22]. This study is unique in studying experimentally ageing stereotypes. The effects of manipulating age information show that more research of this type is needed.

##  Conflict of Interest

There is no financial involvement by any of the authors in this work.

## Figures and Tables

**Table 1:** Ratings of physicians, means (SD), on the probability (1 = very unlikely, 6 = very likely) of this diagnosis and the need for treatment (1 = very unlikely, 6 = very likely).

Probability of diagnosis	Young	Old	
Depression	4.69 (1.18)	4.50 (0.97)	*T* = 1.5, *P* < 0.14
Dementia	1.99 (0.98)	3.63 (1.16)	*T* = 12.2, *P* < 0.001
Anxiety	2.99 (1.26)	2.74 (1.01)	*T* = 2.2, *P* < 0.032
Somatic illness	2.40 (1.15)	3.40 (0.91)	*T* = 1.9, *P* < 0.055

Needed treatment			

Supportive counselling	3.80 (1.36)	4.22 (1.24)	*T* = 3.1, *P* < 0.003
Referral to psychiatrist	3.84 (1.48)	3.16 (1.40)	*T* = 4.7, *P* < 0.001
Referral to inpatient care	2.23 (1.14)	1.99 (0.97)	*T* = 2.0, *P* < 0.053
Psychopharmaca	4.26 (1.08)	4.02 (1.02)	*T* = 2.1, *P* < 0.038
Psychotherapy	4.79 (0.99)	3.01 (1.22)	*T* = 13.0, *P* < 0.001
